# Rising Prevalence of Mild Chronic Gastritis in Children: A Single Center Experience

**DOI:** 10.1177/10935266241238625

**Published:** 2024-03-28

**Authors:** Rohit Josyabhatla, Mary Lauren Wood, Amber Gafur, Nina Tatevian, Amanda S. Tchakarov, Syed Shahrukh Hashmi, Jon Marc Rhoads, Melissa Renee Van Arsdall

**Affiliations:** 1 Division of Gastroenterology, Hepatology and Nutrition, Department of Pediatrics, The University of Texas Health Science Center at Houston, McGovern Medical School, Houston, TX, USA; 2 Kids GI Kare, Cypress, TX, USA; 3 Department of Pediatrics, The University of Texas Health Science Center at Houston, McGovern Medical School, Houston, TX, USA; 4 Department of Pathology and Laboratory Medicine, The University of Texas Health Science Center at Houston, McGovern Medical School, Houston, TX, USA; 5 Pediatrics Research Center, Department of Pediatrics, The University of Texas Health Science Center at Houston, McGovern Medical School, Houston, TX, USA

**Keywords:** abdominal pain, esophagitis, endoscopy, biopsy, gastritis, vomiting

## Abstract

**Objectives and Methods:**

We analyzed upper endoscopic and histological findings in 3 cohorts of children undergoing upper gastrointestinal endoscopy over a 10-year period. Five hundred seventy-nine patients were identified, with 244 (42%), 199 (35%), and 136 (23%) in the 2011, 2015, and 2019 cohorts, respectively. The most common symptoms and signs were abdominal pain, vomiting, failure to thrive, and diarrhea.

**Results:**

The number of patients who had histological evidence of chronic gastritis increased from 2011 (n = 70, 29%) to 2015 (n = 106, 53%) and 2019 (n = 92, 68%; *P* < .001). The prevalence of “normal” endoscopic gastric findings was higher in controls (n = 247, 90%) compared to cases (n = 201, 76%; *P* < .001). There was a small but statistically significant difference in endoscopic esophageal grading (*P* = .008) over time, with lower grades being more prevalent in 2011 compared to 2015 (*P* = .026) and 2019 (*P* = .001). Crude comparisons of the predictors (sex, weight percentile, payor type, month of endoscopy, symptom duration, PPI exposure, and endoscopic stomach findings) yielded no difference between cases and controls.

**Conclusions:**

There has been a significant rise in the prevalence of mild chronic gastritis or non-specific gastritis over the last decade in our population.

## Introduction

Gastritis is a histological diagnosis, characterized by the presence of inflammatory cells, identified on endoscopic gastric biopsies; gastropathy, on the other hand, is a term used to describe abnormalities in the gastric mucosa that can be identified on endoscopy, with or without histological changes.^1^ The most common cause of chronic gastritis in children is Helicobacter pylori infection. Other less frequent causes include systemic stress, bile reflux, non-steroid anti-inflammatory drug use, Crohn’s disease, eosinophilic disease, autoimmune gastritis, lymphocytic gastritis, and rarely Zollinger-Ellison Syndrome.^2^

The Sydney System of classification and grading of gastritis, first created in 1994 and subsequently upgraded in 1999, provides pathologists with a framework to review gastric biopsies in a reproducible manner.^3,4^ However, due to the lack of a universally accepted number of mononuclear inflammatory cells in the gastric mucosa, the definition of chronic inflammation is vague.^3^ This has resulted in the use of interchangeable terms like “mild chronic gastritis” (MCG) or “non-specific gastritis” (NSG), which are often noted on histopathology reports, but have questionable clinical relevance.^5^

At our institution, we collectively review pediatric gastrointestinal biopsies, and we have noted a rise in the number of pediatric patients receiving a diagnosis of MCG or NSG on gastric biopsy samples obtained via esophagogastroduodenoscopy (EGD). Attempting to confirm our suspicion, we conducted this retrospective study with a primary aim of calculating the prevalence of MCG/NSG over a 10-year period. We then performed a case-control analysis, with patients who received a diagnosis of MCG/NSG as cases and patients with normal histology as controls, to assess for possible risk factors that might contribute to MCG/NSG.

## Methods

### Design

We performed a retrospective review of 3 cohorts of pediatric EGD cases between August 1 to July 31 for years 2011–2012, 2015–2016, and 2019–2020. This was a sample designed to capture data over a 10-year period, accounting for seasonal variations and recent trends. We collected data from our electronic medical record system. We manually reviewed charts for every patient who underwent an EGD evaluation during the aforementioned time periods and collected information on patient demographics, proton pump inhibitor (PPI) use, indication for endoscopy, gross endoscopic findings, histopathology results, and final dia-gnosis. Our pathologists define MCG/NSG as multifocal (greater than 2) aggregates of 4–8 plasma cells, with or without associated lymphocytes within the lamina propria, without an identifiable cause, such as H. pylori infection (Supplemental Figure 1). For the duration of our study, the biopsies were reviewed by 2 pathologists at our institute, using these criteria for reporting MCG/NSG.

### Inclusion and Exclusion Criteria

Those with biopsy report findings of MCG/NSG, based on our reviewing pathologists’ predefined criteria, were included. Patients who underwent EGD for foreign body removal, who had a prior history of gastrointestinal surgery, who were found to have findings consistent with H. pylori infection, celiac disease, eosinophilic esophagitis/gastroenteritis, or inflammatory bowel disease (IBD) were excluded. Additionally, patients with histologic evidence of activity with the presence of neutrophils (e.g., active gastritis and chronic active gastritis), with more marked chronic inflammation with large clusters or sheets of plasma cells (e.g., chronic inactive gastritis), and those meeting less than the aforementioned histopathological criteria for MCG/NSG (e.g., minimal and superficial chronic inactive gastritis) were excluded (Supplemental Figure 1).

### Outcomes

Our primary outcome measure was the prevalence of MSG/NSG in each group. Our secondary outcome measures were sex, insurance payor type, BMI percentile, and PPI use assessed as risk factors for MSG/NSG.

### Statistical Analysis

Data were reported as frequency and percentages if categorical and as median with interquartile ranges (IQR) if continuous. All the continuous data in this study were not normally distributed. We summarized and compared these characteristics between the 3 groups. Crude comparisons of categorical variables across other categories were performed using a Fisher exact test. Kruskal-Wallis test (with *post-hoc* Dunn’s test) were used to compare the distributions of the continuous variables across groups. Multivariate logistic regression models were utilized to calculate the odds that chronic gastritis (the primary outcome and classified as a “case” in the case-control analysis) had independently increased over time, after adjusting for other factors. Results were reported as odds ratios (OR) and 95% confidence intervals (CI). All analysis was performed in Stata (v.16, College Station, TX). Statistical significance was assumed at a Type I error rate of 5%.

## Results

### Demographics

A total of 579 patients met the inclusion criteria. Across the cohorts, we analyzed endoscopic findings and biopsies on 244 (42%), 199 (35%), and 136 (23%) patients for the years 2011, 2015, and 2019, respectively (Table 1). There was no difference in sex distribution (females n = 299, 52%,) or BMI percentile (median = 58%ile, IQR = 19–86) between the 3 cohorts. Although the majority of the patients had private insurance coverage in each of the cohorts, the proportion was modestly but significantly higher in 2011 (n = 166, 68%) compared to 2015 (n = 111, 56%) and 2019 (n = 81, 60%; *P* = .001).

**Table 1.** Patient Demographics.

	Year			*P*-value
Variable	2011	2015	2019	
Number of patients	244	199	136	
Female, n (%)	119 (49)	104 (52)	76 (56)	.403
Insurance, n (%)				
Private	166 (68)	111 (56)	81 (60)	.001
Public	67 (28)	87 (44)	53 (39)	
Other	10 (4)	1 (1)	2 (1)	
Month, median (IQR)	7 (4–10)	6 (3–10)	8 (6–10)	.001
Month, n (%)				
January	13 (5)	11 (6)	4 (3)	.001
February	23 (9)	15 (8)	10 (7)	
March	21 (9)	27 (14)	9 (7)	
April	23 (9)	15 (8)	1 (1)	
May	18 (7)	21 (11)	4 (3)	
June	21 (9)	16 (8)	12 (9)	
July	18 (7)	14 (7)	11 (8)	
August	28 (11)	17 (9)	19 (14)	
September	15 (6)	7 (4)	15 (11)	
October	22 (9)	22 (11)	18 (13)	
November	18 (7)	13 (7)	17 (13)	
December	24 (10)	21 (11)	16 (12)	
BMI percentile, median (IQR)	59 (24–87)	59 (16–86)	50 (15–86)	.360
PPI use, n (%)	144 (59)	115 (58)	56 (41)	.002
Duration of symptoms (months), n (%)				
<1	8 (3.5)	16 (8.0)	6 (4.7)	.143
1–6	83 (36.6)	44 (22.1)	46 (35.9)	
7–12	38 (16.7)	32 (16.1)	17 (13.3)	
>12	98 (43.2)	107 (53.8)	59 (46.1)	

The number of pediatric EGD cases were uniformly distributed throughout the year for 2011 and 2015 but skewed to a greater number of patients in the latter half of the year for 2019 (71% from July to December in 2019 compared to 51% and 47% in 2011 and 2015, respectively; *P* = .001). PPI use was high all years but less prevalent in 2019 (n = 56, 41%) compared to the earlier years (combined n = 259, 59%; *P* = .002). However, we found that there was no difference in the rate of PPI use in cases compared to controls (53% vs 57%, adjusted OR = 0.92, CI = 0.62–1.37).

### Indications for EGD

The indications for EGD were recorded and ranked for each cohort and compared (Table 2). The most common indi-cation remained constant over the 10-year period, with abdominal pain being listed in at least half of the patients in each cohort. Other common causes were vomiting (second most common in 2011 and 2015, fourth in 2019) and failure to thrive (third most common in 2011 and 2015, second in 2019). Diarrhea was the fourth most common indication in 2011, but compared to other indications, was not as prevalent in 2015 (ranked 11th) and 2019 (ranked 9th).

**Table 2.** Indications for Endoscopy.

	Counts, n (%)				Ranks		
Indication	All	2011	2015	2019	2011	2015	2019
Abdominal pain	311 (54)	141 (58)	100 (50)	70 (51)	1	1	1
Vomiting	101 (17)	55 (23)	18 (9)	28 (21)	2	4	2
Failure to thrive	90 (15)	49 (20)	26 (13)	15 (11)	3	2	3
Reflux	53 (9)	19 (8)	21 (10)	13 (10)	5	3	4
Feeding difficulty	31 (5)	18 (7)	4 (2)	9 (7)	6	8	5
Diarrhea	31 (5)	25 (10)	1 (0)	5 (4)	4	11	8
Dysphagia	25 (4)	13 (5)	5 (2)	7 (5)	8	6	6
Nausea	21 (4)	15 (6)	2 (1)	4 (3)	7	10	9
Screening/other	16 (3)	2 (1)	8 (4)	6 (4)	9	5	7
Blood in stool	10 (2)	1 (0)	5 (2)	4 (3)	10	6	9
Hematemesis	8 (1)	0 (0)	4 (2)	4 (3)	12	8	9
Anemia	4 (1)	1 (0)	1 (0)	2 (1)	10	11	12

### Clinical Diagnosis

The final diagnosis was recorded based on a review of out-patient records from the clinic visit following EGD (Supp-lemental Table 1). There was no follow-up visit recorded in many of the patients in each of the 3 cohorts (33%, 25%, and 41% in 2011, 2015, and 2019, respectively). About 25% of all cases in each cohort were given a diagnosis of functional abdominal pain or IBS. The next most common diagnosis was “reflux/dysphagia/dyspepsia.”

### Gross Grading

Gross endoscopic findings of the stomach were stratified at 3 levels: 0—normal, 1—erythema/ulcer, and 2—other (Table 3). Approximately 84% of the patients across all years had normal endoscopic findings. There was no statistically significant difference in gross grading of the stomach findings over the years studied.

**Table 3.** Gross Endoscopic Grading of the Stomach.

Grade	Number of patients, n (%)			*P*-value*
	2011	2015	2019	
0	200 (82)	170 (85)	114 (84)	.642
1	24 (10)	13 (7)	16 (12)	
2	20 (8)	16 (8)	6 (4)	

Grade 0—normal, 1—erythema/ulcer, and 2—other.

* *P*-values from Kruskal Wallis tests for “grading” and chi-square test for “stomach findings.”

### Histopathology

**Figure 1.**
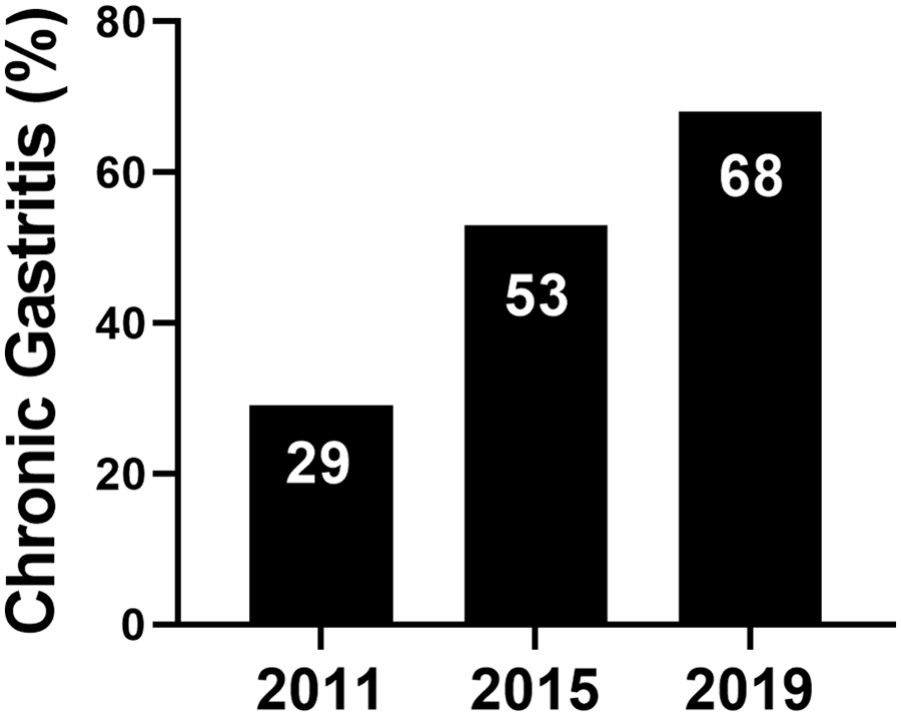
Rise in prevalence of mild chronic gastritis across the 3 cohorts (*P* < .001).

The number of patients who had histological evidence of MCG/NSG increased markedly from 2011 (n = 70, 29%) to 2015 (n = 106, 53%) and 2019 (n = 92, 68%; *P* < .001; Figure 1).

### Comparative Analysis

A total of 539 patients had stomach biopsy results available. These patients were classified as either cases (presence of MCG/NSG on biopsy, n = 265, 46%) or controls (normal histology, n = 274, 47%).

### Crude Analysis

Crude comparisons of the predictors (sex, weight percentile, payor type, month for EGD, symptom duration, PPI exposure, and gross endoscopic stomach findings) yielded no difference between cases and controls except for gross endoscopic stomach findings (Table 4). The prevalence of normal findings was higher in controls (n = 247, 90%) compared to cases (n = 201, 76%; *P* < .001).

**Table 4.** Crude and Adjusted Comparisons of Various Predictors Between Cases and Controls.

	Control	Case	Crude		Adjusted	
Predictor			OR	95% CI	OR	95% CI
Female, n (%)	137 (50)	145 (55)	1.21	0.86–1.70	1.15	0.78–1.69
Insurance, n (%)						
Private	178 (65)	153 (58)	Ref		Ref	
Public	88 (32)	107 (40)	1.41	0.99–2.02	1.27	0.85–1.90
Other	7 (3)	5 (2)	0.83	0.26–2.67	1.57	0.43–5.69
Month of EGD, median (IQR)	7 (3–10)	7 (4–10)	1.01	0.96–1.06	0.98	0.92–1.04
Weight percentile, median (IQR)	58 (19–91)	57 (17.5–84)	1.00	1.00–1.00	1.00	0.99–1.00
PPI use, n (%)	156 (57)	140 (53)	0.84	0.60–1.18	0.92	0.62–1.37
Duration of symptoms (months), n (%)						
<1	11 (4)	19 (7)	1.64	0.73–3.67	1.21	0.50–2.94
1–6	78 (30)	82 (32)	Ref		Ref	
7–12	49 (19)	33 (13)	0.64	0.37–1.10	0.53	0.29–0.97
>12	119 (46)	126 (48)	1.01	0.68–1.50	0.85	0.54–1.34
Gross endoscopic stomach findings, n (%)						
Normal	247 (90)	201 (76)	Ref		Ref	
Erythema/ulcer	15 (5)	37 (14)	3.03	1.62–5.68	3.74	1.84–7.62
Other	12 (4)	27 (10)	2.76	1.37–5.60	4.03	1.87–8.66
Year of EGD, n (%)						
2011	161 (58)	70 (26)	Ref		Ref	
2015	83 (30)	106 (40)	2.94	1.97–4.39	3.22	2.06–5.04
2019	35 (13)	89 (34)	5.85	3.61–9.46	6.62	3.86–11.4

Abbreviations: CI, confidence interval; OR, odds ratio; Ref, referent category.

### Adjusted Analysis

Adjusted logistic regression models were constructed using predictors: sex, weight percentile, payor type, month for EGD, symptom duration, PPI exposure, and gross endoscopic stomach findings. These adjusted models identified independent effects of higher odds of being a case by: (a) cohort year (OR_2015_ = 3.22, 95% CI = 2.06–5.04; OR_2019_ = 6.62, 95% CI = 3.86–11.37, compared to the reference 2011 cohort) and (b) gross endoscopic stomach findings (OR_erythema/ulcer_ = 3.74, 95% CI = 1.84–7.62 compared to patients with normal findings); and independent effects of lower odds of being a case by duration of symptoms (OR_7–12months_ = 0.53, 95% CI = 0.29–0.97 compared to patients who have had symptoms for 1–6 months).

## Discussion

Although commonly noted on histopathology reports, the true prevalence of mild chronic gastritis/ nonspecific gastritis in the pediatric literature has not been reported. In 2 studies that analyzed the role of PPI in NSG, the prevalence of MCG/NSG ranged from 24% to 38%.^6,7^ Our results indicate that there has been a significant rise in the prevalence of MCG/NSG over the last decade at our center, with a prevalence of 68% in 2019–2020, which was independent of H. pylori infection, celiac disease, eosinophilic esophagitis/gastroenteritis, celiac disease, or inflammatory bowel disease. While the diagnosis of MCG/NSG may be subjective, with variations in diagnosis between different pathologists and different institutions, our pathologists used the same criteria for reporting MCG/NSG throughout the study period, strengthening our finding of an increase in this entity at our institution over the last decade.

The risk factors for and clinical implications of MCG/NSG remain unknown. To account for possible socio-economic factors contributing to the rise in prevalence of MCG/NSG, we assessed the trends and differences in insurance payor type. Although there was a trend toward a higher proportion of patients being on public insurance in our 2015 and 2019 cohorts, the majority of patients across all 3 cohorts were on private insurance. Moreover, while both the prevalence of MCG/NSG and proportion of patients with public insurance increased from 2011 to 2015, there was a smaller proportion with public insurance in 2019 versus 2015, despite there being a higher prevalence of MCG/NSG in 2019 compared to 2015. Furthermore, on adjusted logistic regression, there was no statistical significance between the cases and controls based on payor type.

Sleeve gastrectomy specimens provide a unique opportunity to review the spectrum of chronic gastritis and other histopathology in obese patients. In a study from Kuwait on 656 obese patients who underwent sleeve gastrectomy, the prevalence of chronic gastritis was 74.4%.^8^ Only 7.3% of participants were H. pylori positive, indicating a high prevalence of non-H. pylori chronic gastritis in obese adults. However, in another large retrospective study in adults from Australia, the prevalence of NSG was only about 7.2% in 1463 consecutive gastrectomy specimens.^9^ Another study from the USA aimed to better characterize the differences in chronic gastritis in gastric fundic specimens from patients undergoing sleeve gastrectomy between 1996 and 2011. Biopsies from 53/68 (78%) patients, despite being negative for H. pylori, increased plasma cells and lymphoid aggregates. The investigators of that study speculated that these findings could represent a spectrum of normal histology, rather than MCG/NSG.^5^ Despite the increased prevalence of MCG/NSG over time, we did not find a relationship between BMI and MCG/NSG in our population.

PPI use in children can be associated with NSG.^6^ In a previous study at our institute, patients with MCG/NSG were found to have increased odds of PPI use for more than 6 weeks.^7^ We re-evaluated this in the present study, over a longer period with more patients. In our study, PPI use was relatively similar in 2011 and 2015 and slightly less in 2019, despite the increased prevalence of MCG/NSG from 2011 to 2015 and from 2015 to 2019. We also found that there was no difference in the rate of PPI use in cases compared to controls (~55% in each group).

Of note, the retrospective nature of our study did not allow us to analyze some other variables that could potentially contribute to MCG/NSG. For example, non-steroid anti-inflammatory drug (NSAID) use is difficult to capture accurately on chart review, because NSAIDs are over-the-counter medications, with use not consistently reported in medical charts. Similarly, while environmental conditions, dietary factors, preceding antibiotic use, and actual adherence to PPI treatment could have potentially contributed to the increased prevalence of MCG/NSG over the last decade at our institution, these variables could not be adequately assessed with the records available for review. Also, the long-term outcome of these patients could not be determined.

Indications for upper endoscopy remained similar across the years, with abdominal pain, vomiting, failure to thrive, and reflux being the most common (Table 2). This is consistent with common indications for endoscopy in pediatrics, and we could not ascertain a link between the indication and MCG/NSG.^10^ We also analyzed the diagnosis that these patients were assigned, based on the first outpatient visit post the endoscopy. Over the years, the most common diagnosis was functional abdominal pain/IBS or unknown (did not have a follow up in clinic). This again raises the question of whether MCG/NSG is a disease.

To our knowledge, this is the first study to report the rising prevalence of MCG/NSG over a long timeframe, including recent trends. Our analysis of the role of previously described risk factors, including obesity and PPI use, in addition to insurance payor type, did not find a relationship with the rising prevalence of MCG/NSG. Further studies are therefore required to confirm the increasing prevalence of MCG/NSG and to assess other potential risk factors across different geographic regions.

## Conclusion

The last decade has seen a significant rise in the prevalence of pediatric mild chronic gastritis at our institute. There is a need to better characterize this entity as either a normal variant or a disease process that requires further characterization.

## Supplemental Material

sj-docx-1-pdp-10.1177_10935266241238625 – Supplemental material for Rising Prevalence of Mild Chronic Gastritis in Children: A Single Center Experience Supplemental material, sj-docx-1-pdp-10.1177_10935266241238625 for Rising Prevalence of Mild Chronic Gastritis in Children: A Single Center Experience by Rohit Josyabhatla, Mary Lauren Wood, Amber Gafur, Nina Tatevian, Amanda S. Tchakarov, Syed Shahrukh Hashmi, Jon Marc Rhoads and Melissa Renee Van Arsdall in Pediatric and Developmental Pathology

sj-docx-2-pdp-10.1177_10935266241238625 – Supplemental material for Rising Prevalence of Mild Chronic Gastritis in Children: A Single Center Experience Supplemental material, sj-docx-2-pdp-10.1177_10935266241238625 for Rising Prevalence of Mild Chronic Gastritis in Children: A Single Center Experience by Rohit Josyabhatla, Mary Lauren Wood, Amber Gafur, Nina Tatevian, Amanda S. Tchakarov, Syed Shahrukh Hashmi, Jon Marc Rhoads and Melissa Renee Van Arsdall in Pediatric and Developmental Pathology
