# Supplementary material for: Rising Prevalence of Mild Chronic Gastritis in Children: A Single Center Experience
Source: Pediatr Dev Pathol. 2024 Mar 28;27(3):235–40. doi: 10.1177/10935266241238625 (PMC11088214; doi:10.1177/10935266241238625)
Supplement: sj-docx-1-pdp-10.1177_10935266241238625 – Supplemental material for Rising Prevalence of Mild Chronic Gastritis in Children: A Single Center Experience [file sj-docx-1-pdp-10.1177_10935266241238625.docx]

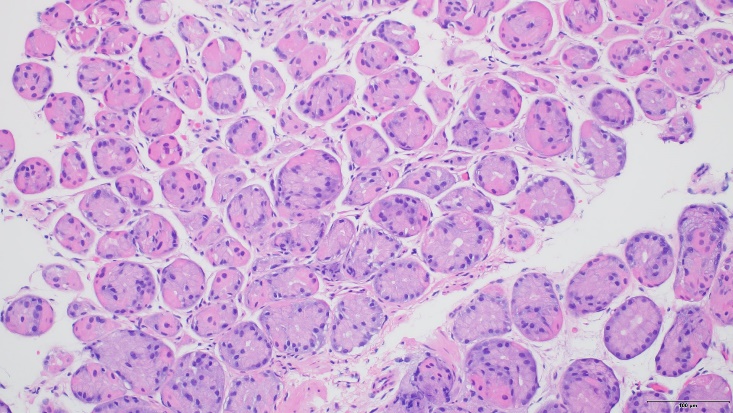

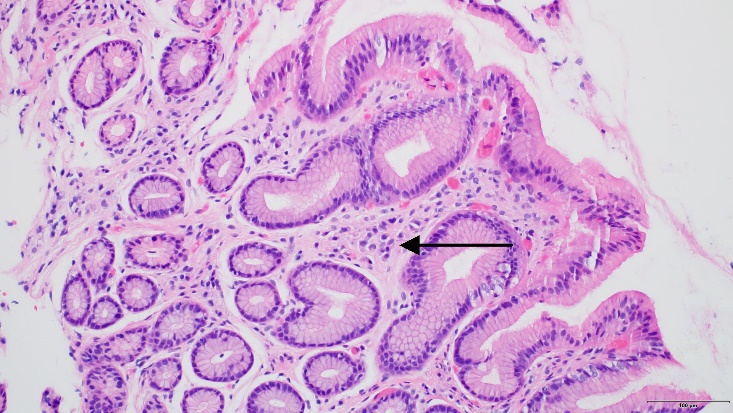

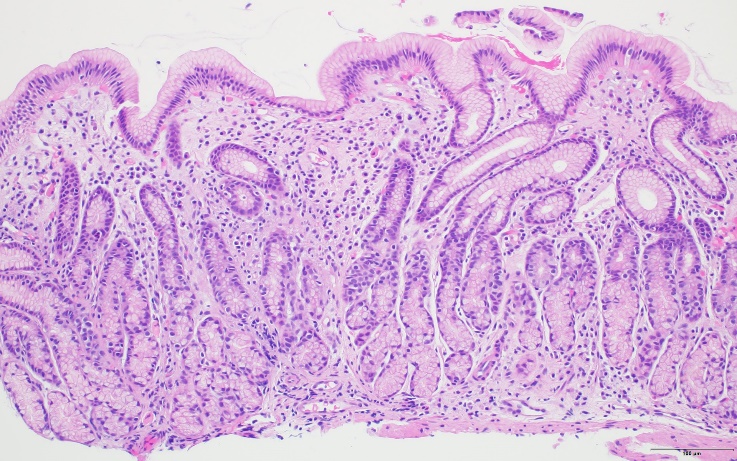

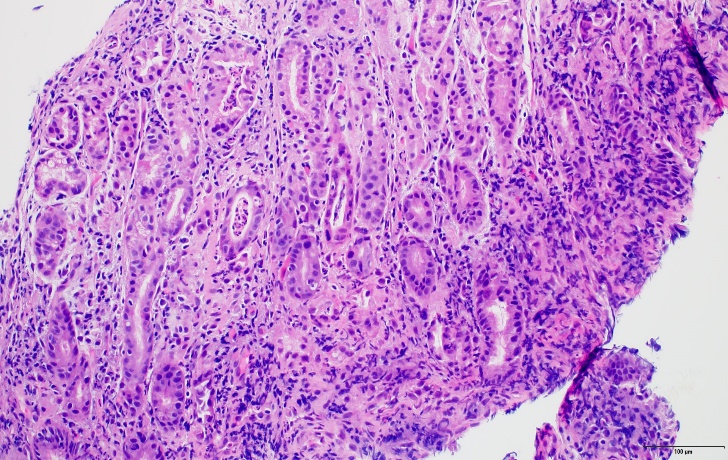


A

D

C

B

Supplementary Figure 1. Grading of inflammation within gastric mucosa at our institution. A (H&E, 20X) normal gastric mucosa with limited inflammatory infiltrate within the lamina propria. B (H&E, 20X) mild chronic gastritis with clusters of 4-8 plasma cells (arrow). C (H&E, 20X) moderate chronic gastritis with numerous plasma cells within the lamina propria (associated with H. pylori in this case). D (H&E, 20X) chronic active gastritis with lymphocytes, plasma cells, and neutrophils within the lamina propria along with cryptitis and crypt abscess formation.
