# Supplementary material for: Rising Prevalence of Mild Chronic Gastritis in Children: A Single Center Experience
Source: Pediatr Dev Pathol. 2024 Mar 28;27(3):235–40. doi: 10.1177/10935266241238625 (PMC11088214; doi:10.1177/10935266241238625)
Supplement: sj-docx-2-pdp-10.1177_10935266241238625 – Supplemental material for Rising Prevalence of Mild Chronic Gastritis in Children: A Single Center Experience [file sj-docx-2-pdp-10.1177_10935266241238625.docx]

| **Supplemental Table 1. Final clinical diagnosis** |  |  |  |
| --- | --- | --- | --- |
| n (%) | 2011 | 2015 | 2019 |
| Esophageal reflux/dyspepsia/dysphagia | 44 (18) | 31 (16) | 16 (12) |
| Functional Abdominal Pain / IBS | 62 (25) | 55 (28) | 34 (25) |
| Abdominal Migraines / Cyclic Vomiting | 1 (0) | 5 (2) | 4 (3) |
| Inadequate Caloric Intake | 24 (10) | 14 (7) | 5 (4) |
| Disaccharidase Deficiency | 2 (1) | 7 (4) | 9 (6) |
| Other | 31 (13) | 37 (18) | 12 (9) |
| Unknown | 80 (33) | 50 (25) | 56 (41) |
